# Low- vs High-Dose 5-FU in Triplet Chemotherapy Plus Bevacizumab for Patients With Colorectal Cancer

**DOI:** 10.1001/jamanetworkopen.2024.24855

**Published:** 2024-07-31

**Authors:** William J. Chapin, Wei-Ting Hwang, Ronac Mamtani, Mark H. O’Hara

**Affiliations:** 1 Division of Hematology and Oncology, Department of Medicine, Perelman School of Medicine at the University of Pennsylvania, Philadelphia; 2 Department of Biostatistics, Epidemiology, and Informatics, Perelman School of Medicine at the University of Pennsylvania, Philadelphia

## Abstract

This cohort study examines the association of low-dose vs high-dose 5-fluorouracil with overall survival when used in triplet chemotherapy plus bevacizumab in patients from the metastatic colorectal cancer (CRC).

## Introduction

Standard first-line systemic therapy for metastatic colorectal cancer (mCRC) includes doublet chemotherapy (5-fluorouracil plus either oxaliplatin or irinotecan) plus bevacizumab or antiepidermal growth factor receptor (EGFR) therapy.^1^ For patients receiving chemotherapy plus bevacizumab who have an excellent performance status, several randomized clinical trials demonstrated that triplet chemotherapy (5-fluorouracil, oxaliplatin, and irinotecan) plus bevacizumab (TripletBev) improved overall survival (OS) compared with doublet chemotherapy plus bevacizumab, albeit with increased rates of neutropenia and diarrhea.^2,3^ These trials used high-dose continuous-infusion 5-fluorouracil (3200 mg/m^2^ per 48 hours); however, national guidelines suggest using low-dose continuous-infusion 5-fluorouracil (2400 mg/m^2^ per 46 hours) due to lower tolerance in patients, which is thought to be secondary to regional variation in folate consumption.^1,4^ Because dosing strategies have not been directly compared, we examined the association of low-dose vs high-dose 5-fluorouracil with OS when used in TripletBev in patients from the US with mCRC.

## Methods

This retrospective cohort study included patients with mCRC receiving first-line TripletBev. The study was deemed exempt from review and consent by the institutional review board at the University of Pennsylvania and followed the Strengthening the Reporting of Observational Studies in Epidemiology (STROBE) reporting guideline. We used the Flatiron Health database, which is a longitudinal database comprising deidentified patient-level structured and unstructured data curated via technology-enabled abstraction.^5,6^ During the study, the deidentified data originated from approximately 280 cancer clinics and 800 sites of care. We excluded records missing height, weight, or dosing information at the time of the first administration (eMethods and eFigure in Supplement 1).

Patients were classified as receiving a low-dose (2400 mg/m^2^ infusion ±10%) or high-dose 5-fluorouracil (3200 mg/m^2^ infusion ±10%) as part of first administration of TripletBev as first-line therapy with OS as the outcome. Prespecified variables considered as potential confounders are detailed in the eMethods and eTable in Supplement 1). Statistical analyses were performed in Stata version 16.1 (StataCorp) and were 2-sided with α = .05. Missing values were addressed with multiple imputation with chained equations. Prespecified covariates with an imbalance between low-dose and high-dose 5-fluorouracil groups, defined by a standardized difference of less than −0.10 or more than 0.10, were included in a multivariable Cox proportional hazards model to evaluate the adjusted association of low- vs high-dose 5-fluorouracil with OS (eMethods in Supplement 1). A post hoc analysis that included patients receiving triplet chemotherapy with or without bevacizumab was performed using the same methods. Data were analyzed from August 1, 2023, to May 2, 2024.

## Results

Between October 23, 2014, and October 31, 2022, 255 patients (157 males [62%] and 98 females [38%]) with a median (IQR) age of 52.0 (45.0-61.0) years met the inclusion criteria. They received TripletBev, with 184 patients (72%) receiving low-dose 5-fluorouracil and 71 patients (28%) receiving high-dose 5-fluorouracil (Table). Key differences between treatment groups included the year of metastatic diagnosis and the use of 5-fluorouracil bolus (Table). Median OS was 34.2 (95% CI, 23.1-45.4) months for low-dose and 34.0 (95% CI, 24.0-49.6) months for high-dose 5-fluorouracil (HR, 1.04; 95% CI, 0.69-1.56) (*P* = .87). There was no association between low-dose vs high-dose 5-fluorouracil and OS in the multivariable analysis (Figure).

**Table.** Baseline Characteristics for Patients Who Received Triplet Chemotherapy Plus Bevacizumab

Characteristics	Patients, No. (%)			*P* value^a^	Standardized difference^b^
	Total (N = 255)	High-dose 5-FU (n = 71)	Low-dose 5-FU (n = 184)		
Age, median (IQR)	52.0 (45.0-61.0)	52.0 (44.0-59.0)	52.0 (45.0-61.0)	.92	−0.03
Gender					
Men	157 (62)	40 (56)	117 (64)	.29	0.15
Women	98 (38)	31 (44)	67 (36)		
Race or ethnicity					
Asian	10 (4)	1 (1)	9 (5)	.08	0.20
Hispanic or Latinx	24 (9)	9 (13)	15 (8)		−0.15
Non-Hispanic Black	24 (9)	9 (13)	15 (8)		−0.15
Non-Hispanic White	155 (61)	47 (66)	108 (59)		−0.15
Other race^c^	21 (8)	2 (3)	19 (10)		0.31
Unknown	21 (8)	3 (4)	18 (10)		0.22
Insurance status					
Commercial	185 (73)	53 (75)	132 (72)	.96	−0.07
Medicaid	11 (4)	3 (4)	8 (4)		0.01
Medicare	10 (4)	2 (3)	8 (4)		0.08
Other	14 (5)	3 (4)	11 (6)		0.08
No documented insurance	35 (14)	10 (14)	25 (14)		−0.01
Practice type					
Academic	77 (30)	23 (32)	54 (29)	.63	0.07
Community	178 (70)	48 (68)	130 (71)		
Primary tumor sidedness					
Right-sided	60 (24)	21 (30)	39 (21)	.36	0.18
Left-sided	154 (60)	39 (55)	115 (63)		
Missing	41 (16)	11 (15)	30 (16)		
Metastatic disease at diagnosis					
Synchronous	216 (85)	60 (85)	156 (85)	.96	−0.01
Metachronous	39 (15)	11 (15)	28 (15)		
Year of metastatic diagnosis					
2014-2018	66 (26)	33 (46)	33 (18)	<.001	0.64
2019-2022	189 (74)	38 (54)	151 (82)		
MMR/MSI					
MMRp/MSS	213 (84)	53 (75)	160 (87)	.060	−0.15
MMRd/MSI-H	7 (3)	3 (4)	4 (2)		
Missing	35 (14)	15 (21)	20 (11)		
RAS/RAF status					
Mutation negative	80 (31)	19 (27)	61 (33)	.39	−0.06
Mutation positive	120 (47)	33 (46)	87 (47)		
Missing	55 (22)	19 (27)	36 (20)		
ECOG performance status					
0-1	200 (78)	57 (80)	143 (78)	.76	0.09
≥2	11 (4)	2 (3)	9 (5)		
Missing	44 (17)	12 (17)	32 (17)		
Carcinoembryonic antigen, median (IQR)	44.4 (4.8-278.3)	55.0 (5.3-244.1)	43.3 (4.8-288.5)	.78	−0.04
Bilirubin, median (IQR)	0.4 (0.3-0.6)	0.4 (0.3-0.6)	0.4 (0.3-0.6)	.38	NA^d^
Albumin, median (IQR)	3.9 (3.6-4.2)	3.9 (3.7-4.2)	3.9 (3.5-4.1)	.66	NA^d^
Creatinine, median (IQR)	0.8 (0.7-0.9)	0.8 (0.6-0.9)	0.8 (0.7-0.9)	.44	0
Irinotecan dose, median (IQR), mg/m^2^	163.0 (156.2-165.8)	164.0 (162.3-166.2)	162.5 (147.9-165.3)	.008	−0.30
Oxaliplatin dose, median (IQR), mg/m^2^	84.2 (82.7-85.3)	84.5 (83.5-85.6)	84.2 (82.2-85.3)	.021	−0.24
5-FU bolus					
No	234 (92)	69 (97)	165 (90)	.051	0.31
Yes	21 (8)	2 (3)	19 (10)		

Abbreviations: ECOG, Eastern Cooperative Oncology Group; 5-FU, 5-fluorouracil; MMRd/MSI-H, mismatch repair deficient/microsatellite instability-high; MMR/MSI, mismatch repair/microsatellite instability status; MMRp/MSS, mismatch repair proficient/microsatellite stable; RAS/RAF status, *KRAS, NRAS,* and *BRAF* status.

^a^ For all categorical variables, *P* value is from Pearson χ^2^ test. For continuous variables, *P* value is from Wilcoxon rank-sum test.

^b^ Standardized differences in means (for continuous variables) or proportions (for binary variables) between treatment groups. For multilevel categorical variables, a binary dummy variable for each level was generated to perform the calculation of standardized difference in proportions between treatment groups.

^c^ Other race includes patients who self-reported their race as American Indian or Alaska Native, Native Hawaiian or Other Pacific Islander, and those who self-reported multiple race categories.

^d^ Balance was assessed on albumin-bilirubin grade rather than on the individual components. The standardized difference in the albumin-bilirubin grade between the treatment groups was 0.09.

**Figure.**
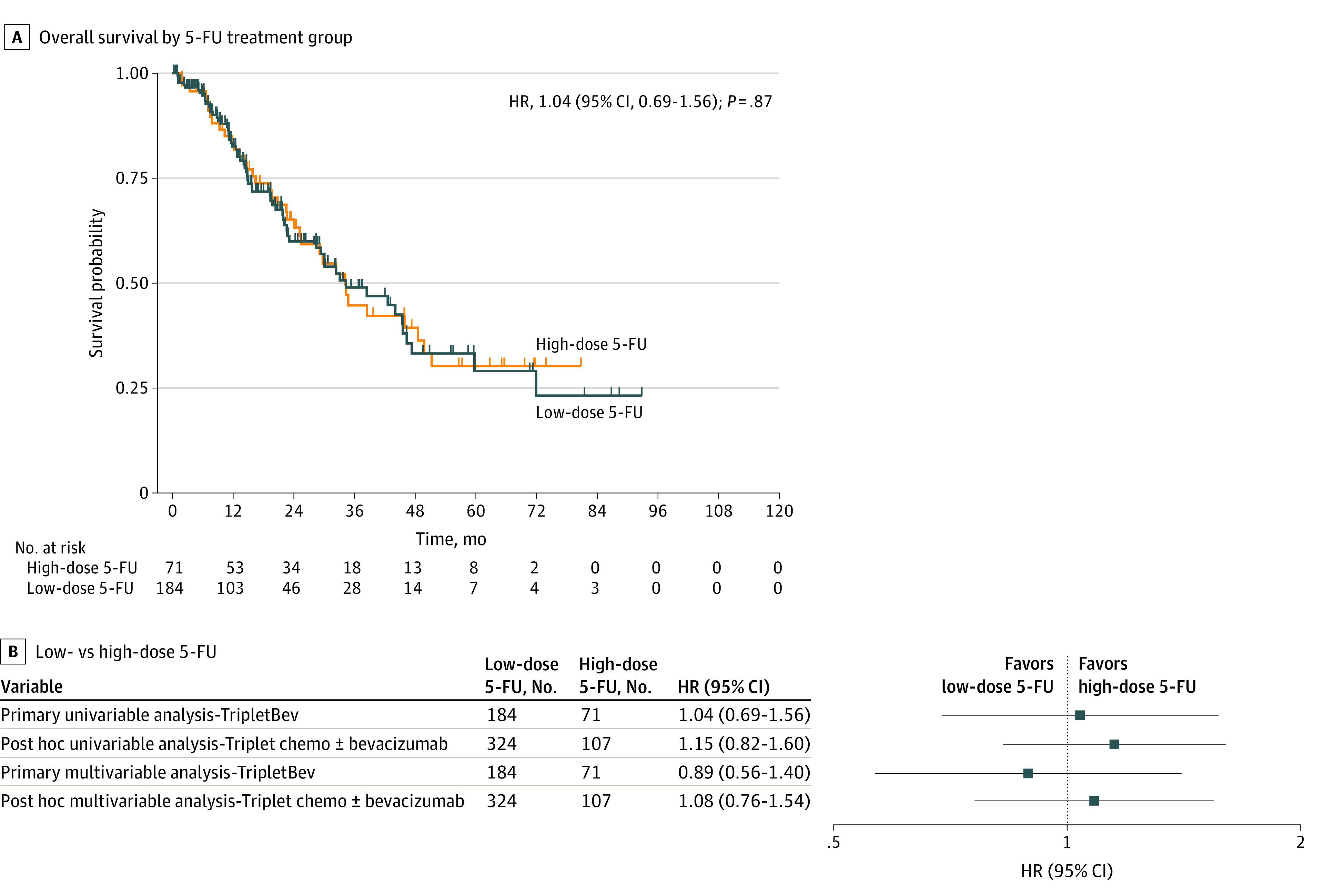
Univariable and Multivariable Analyses of Overall Survival by 5-Fluorouracil (5-FU) Treatment Group A, Kaplan-Meier analysis of overall survival by 5-FU treatment group. Hazard ratio (HR) (low- vs high-dose 5-FU [reference category]), 95% CI, and *P* value are from the univariable Cox proportional hazards model. Median (IQR) follow-up time for patients who were censored was 13 (6.9-23.3) months in the low-dose 5-FU group and 28.4 (15.0-56.9) months in the high-dose 5-FU group. B, Forest plot demonstrating the results for low- vs high-dose 5-FU (reference category) for the primary and post hoc analysis. The primary multivariable model included year of metastatic diagnosis, 5-FU bolus, tumor sidedness, gender, mismatch repair/microsatellite instability status, and oxaliplatin and irinotecan doses due to imbalance on these variables. The post hoc multivariable model included year of metastatic diagnosis, 5-FU bolus, performance status, tumor sidedness, use of bevacizumab, and oxaliplatin and irinotecan dose due to imbalance. TripletBev indicates triplet chemotherapy plus bevacizumab.

## Discussion

In this cohort study of patients with mCRC receiving first-line chemotherapy with TripletBev, we observed no clear association between low-dose vs high-dose 5-fluorouracil with OS. Limitations include limited power to detect potentially clinically meaningful differences between treatment groups. Additionally, we cannot rule out unmeasured confounding and do not have data on additional important patient outcomes, such as toxicity. These results are applicable to patients in the US who are receiving TripletBev. This group represents a small minority of patients receiving systemic therapy for mCRC. These data reassure clinicians and patients who choose to use low-dose 5-fluorouracil and provide the first comparative data supporting clinical practice guidelines, which recommend this approach for US patients receiving TripletBev.

## Notes

Supplement 1.
eMethods.
**eTable.** *International Classification of Diseases, Ninth Revision *and *International Statistical Classification of Diseases and Related Health Problems, Tenth Revision *Diagnostic Codes Used to Define Tumor-Sidedness **eFigure.** Patient Flow Diagram Demonstrating Patient Inclusion and Reasons for Exclusion From the Study
eReferences.

Supplement 2.
Data Sharing Statement
