# Supplementary material for: Low- vs High-Dose 5-FU in Triplet Chemotherapy Plus Bevacizumab for Patients With Colorectal Cancer
Source: JAMA Netw Open. 2024 Jul 31;7(7):e2424855. doi: 10.1001/jamanetworkopen.2024.24855 (PMC11292450; doi:10.1001/jamanetworkopen.2024.24855)
Supplement: Supplement 1. — eMethods. eTable. International Classification of Diseases, Ninth Revision and International Statistical Classification of Diseases and Related Health Problems, Tenth Revision Diagnostic Codes Used to Define Tumor-Sidedness eFigure. Patient Flow Diagram Demonstrating Patient Inclusion and Reasons for Exclusion From the Study eReferences. [file jamanetwopen-e2424855-s001.pdf]

Supplemental Online Content

Chapin WJ, Hwang WT, Mamtani R, O'Hara MH. Low- vs high-dose 5-FU in triplet chemo plus bevacizumab for patients with colorectal cancer. *JAMA Netw Open*. 2024;7(7):e2424855. doi:10.1001/jamanetworkopen.2024.24855

eMethods.

eTable. *International Classification of Diseases, Ninth Revision* and *International Statistical Classification of Diseases and Related Health Problems, Tenth Revision* Diagnostic Codes Used to Define Tumor-Sidedness

eFigure. Patient Flow Diagram Demonstrating Patient Inclusion and Reasons for Exclusion From the Study

eReferences.

This supplemental material has been provided by the authors to give readers additional information about their work.

© 2024 Chapin WJ et al. *JAMA Network Open.*

eMethods

1.1. Detailed inclusion and exclusion criteria:

Inclusion criteria: Patients were included if they had a diagnosis of metastatic colorectal cancer and received their first administration (index-date) of first-line treatment with triplet chemotherapy (5-FU, irinotecan, oxaliplatin, and bevacizumab) from 10/23/2014 – 10/31/22. Patients must have received all chemotherapy agents (5-FU, irinotecan, and oxaliplatin) concurrently on the index-date. Patients could be included if bevacizumab was administered concurrent to the index-date or within the first 60 days following the index-date to acount for the common practice of adding bevacizumab to chemotherapy within several cycles of treatment start.

Exclusion criteria: Patients were excluded if height (closest measurement to index-date from any time prior to index-date to 28-days post-index date), weight (closest to index-date from any time prior to index-date to 28-days post-index-date), or dose (of 5FU, irinotecan, or oxaliplatin on the index-date) were missing. Patients were excluded if there was an absence of all structured data within 90-days following diagnosis of metastatic colorectal cancer in order to exclude patients with potentially misclassified line of therapy data (oncologist-defined, rule-based) due to treatment being received elsewhere. Patients without a continuous infusion 5-FU dose in the range of 2,400mg/m2 +/- 10% or 3,200mg/m2 +/- 10% were excluded.

1.2. Detailed definition of exposure:

Low- versus high-dose continuous infusion 5FU: First, body surface area for each patient was calculated using the height and weight closest to index-date within the window of any time prior to index-date to 28-days post-index-date using the Mosteller formula $(((\text{height in cm} * \text{weight in kg})/3600)^{1/2})$. Dose of each administration of 5-FU on the index-date (some bolus doses and some continuous infusion 5-FU doses) in milligrams was divided by body surface area to give the dose in mg/m^2. Doses less than 600mg/m2 were considered bolus doses while anything greater than 600mg/m2 was considered a potential continuous infusion dose. For the primary exposure, 5-FU continuous infusion doses of 2,400mg/m2 +/- 10% were classified as low-dose continuous infusion 5-FU and doses of 3,200mg/m2 +/- 10% were classified as high-dose continuous infusion 5-FU.

1.3. Detailed definitions of covariates:

Pre-specified covariates considered to be potential confounders included age, gender, year of metastatic diagnosis, baseline carcinoembryonic antigen (CEA), KRAS, NRAS, and BRAF (RAS/RAF) alteration status, mismatch repair deficiency or microsatellite instability status (MMR/MSI status), tumor sidedness, synchronous versus metachronous metastases, Eastern Cooperative Oncology Group (ECOG) performance status, use of 5FU bolus, irinotecan dose, and oxaliplatin dose. Additional covariates including race/ethnicity, academic vs community practice, insurance status, renal dysfunction and (albumin-bilirubin) ALBI grade were evaluated for descriptive purposes.

Age: Defined as the date of first systemic treatment minus birth-year. Coded as a continuous variable.

Gender: Self-reported by patients and recorded as a binary variable (man or woman).

Race/ethnicity: Race and ethnicity were self-reported and recorded in the Flatiron Health electronic health record-derived de-identified database. This variable was coded using the following categories for the purposes of the study: Asian, Hispanic or Latin-X, Non-Hispanic Black, Non-Hispanic White, Other Race, and Unknown. The “Other Race” category included patients who self-reported race/ethnicity as American Indian or Alaska Native, Native Hawaiian or Other Pacific Islander, and those who self-reported multiple race categories.

Academic or community practice: Binary variable defined by practice type of the visit where patient initiated first systemic treatment.

© 2024 Chapin WJ et al. *JAMA Network Open.*

Year of metastatic diagnosis: Binary variable defined by calendar year of diagnosis of metastatic colorectal cancer. Categories included < 2018 and >/= 2018.

Insurance at the time of first-systemic therapy initiation: Categorical variable defined as insurance status closest to the date of first systemic treatment (index date) after excluding insurance entries with: missingness on both start and end dates, a start date after index date, an end date prior to index date, and those coded as patient assistance program or other payers. Categories included commercial insurance, Medicaid, Medicare, Other, and No documented insurance.

Baseline CEA: Continuous variable defined by CEA measurement within 30 days prior to and including the index date. CEA is presented in units of micrograms per L.

RAS/RAF mutation status: Binary variable defined by *KRAS, NRAS,* and *BRAF* mutation status on tumor tissue closest to the index date, but within the window from any time prior to index date to 60 days following index date. Observations were categorized as RAS/RAF wild-type if wild-type for all three genes and as RAS/RAF altered if an alteration was present in any of the three genes, even if information was unavailable for one or both of the other genes. However, if no RAS/RAF alterations were present and there was missing information for at least one of the three genes, then the observation was coded as missing.

Mismatch repair (MMR)/microsatellite instability (MSI) Status: Binary variable defined by MMR/MSI status closest to the index date and occurring within the window of any time prior to index date to within 60 days following the index date. An observation was categorized as MMR deficient or MSI high (MMRd/MSI-H) if either mismatch repair deficiency or microstatellite instability-high status were present. Otherwise, the observation was coded as MMR proficient or microsatellite stable (MMRp/MSS) if not missing.

Tumor sidedness: Binary variable defined by presence of ICD9 or ICD10 codes specific for tumor sidedness, prior to and including the index date. This approach was modeled off that validated by Luhn et al. with several key differences.[1] Rather than choosing only diagnosis codes for colorectal cancer closest to the cancer diagnosis date as was performed in the validation study, we included only colorectal cancer codes that indicated a specific side of the colon (eTable 1) that were present prior to, or including the index date. Amongst these codes, the code closest to the index date was chosen for this determination. The second difference was that we did not include diagnosis codes for the anus or anal canal as sidedness-specific codes for our patients with metastatic colorectal cancer. From the ascending colon the splenic flexure (including all of the transverse colon; excluding splenic flexure) was considered right-sided while from the splenic flexure to rectum was considered left-sided. The specific diagnostic codes to determine sidedness are included in eTable 1.

Synchronous or Metachronous metastatic disease: Binary variable defined by the time from colon or rectal cancer diagnosis to the time of diagnosis of metastatic disease. Less than 90 days was defined as synchronous while greater than or equal to 90 days was defined as metachronous.

ECOG performance status: Binary variable defined as ECOG status recorded within 30 days prior to 7 days after index date. Categories include ECOG 0 – 1 and ECOG >/= 2.

Renal dysfunction: Binary variable assessed using creatinine measured within 30 days prior to, and including, the index date. Categories include creatinine > 1.3 mg/dL and creatinine </= 1.3 mg/dL.

ALBI grade: Categorical variable defined per Johnson et al., 2015.[2]

1.4. Detailed definition of outcome:

Overall survival: Time from first treatment administration (index-date) to death, with censoring for last confirmed activity including in-person visit or confirmation of treatment administration.

© 2024 Chapin WJ et al. *JAMA Network Open.*

1.5. Statistical analysis:

1.5.1. Sample size determination
As this study was retrospective and used and existing dataset, sample size was determined by the number of patients meeting eligibility criteria.

1.5.2. Univariable analysis
Univariable analysis of OS by treatment group was assessed using the Kaplan Meier method with log-rank testing an univariable Cox Proportional Hazards modelling.

1.5.3. Multiple imputation with chained equations
Missing values from pre-specified covariates were assumed to be missing at random (MAR) and multiple imputation with chained equations with 25 imputations was used to minimize bias related to missing covariates. Following multiple imputation of missing covariates, balance of covariates between low- and high-dose 5FU groups was assessed using standardized differences in means for continuous variables and standardized differences in proportions for each level of binary, categorical, or ordinal variables using *pbalchk* in Stata.[3]

1.5.4. Multivariable Cox Proportional Hazards Model
This multivariable model was performed in each of the 25 imputed data sets with the treatment effect estimates combined to obtain the overall treatment effect estimate using Rubin's rules.[4]

2. eTables:

eTable 1: ICD-9 and ICD-10 diagnostic codes used to define tumor-sidedness.

ICD9	Text	ICD10	Text
Right-sided Primary Tumor			
153.0	Malignant Neoplasm of Colon; Hepatic flexure	C18.3	Malignant Neoplasm of Hepatic flexure
153.1	Malignant Neoplasm of Colon; Transverse colon	C18.4	Malignant Neoplasm Transverse colon
153.4	Malignant Neoplasm of Colon; Cecum	C18.0	Malignant Neoplasm of Cecum
153.6	Malignant Neoplasm of Colon; Ascending colon	C18.2	Malignant Neoplasm of Ascending colon
Left-sided Primary Tumor			
153.2	Malignant Neoplasm of Colon; Descending colon	C18.6	Malignant Neoplasm of Descending colon
153.3	Malignant Neoplasm of Colon; Sigmoid colon	C18.7	Malignant Neoplasm of Sigmoid colon
153.7	Malignant Neoplasm of Colon; Splenic flexure	C18.5	Malignant Neoplasm of the Splenic flexure
154.0	Malignant Neoplasm of Rectum, Rectosigmoid Junction, and Anus; Rectosigmoid junction	C19	Malignant Neoplasm of Rectosigmoid junction
154.1	Malignant Neoplastm of Rectum, Rectosigmoid junction, and Anus; Rectum	C20	Malignant Neoplasm of Rectum

© 2024 Chapin WJ et al. *JAMA Network Open.*

3. eFigures:

eFigure 1: Patient flow diagram demonstrating patient inclusion and reasons for exclusion from the study.

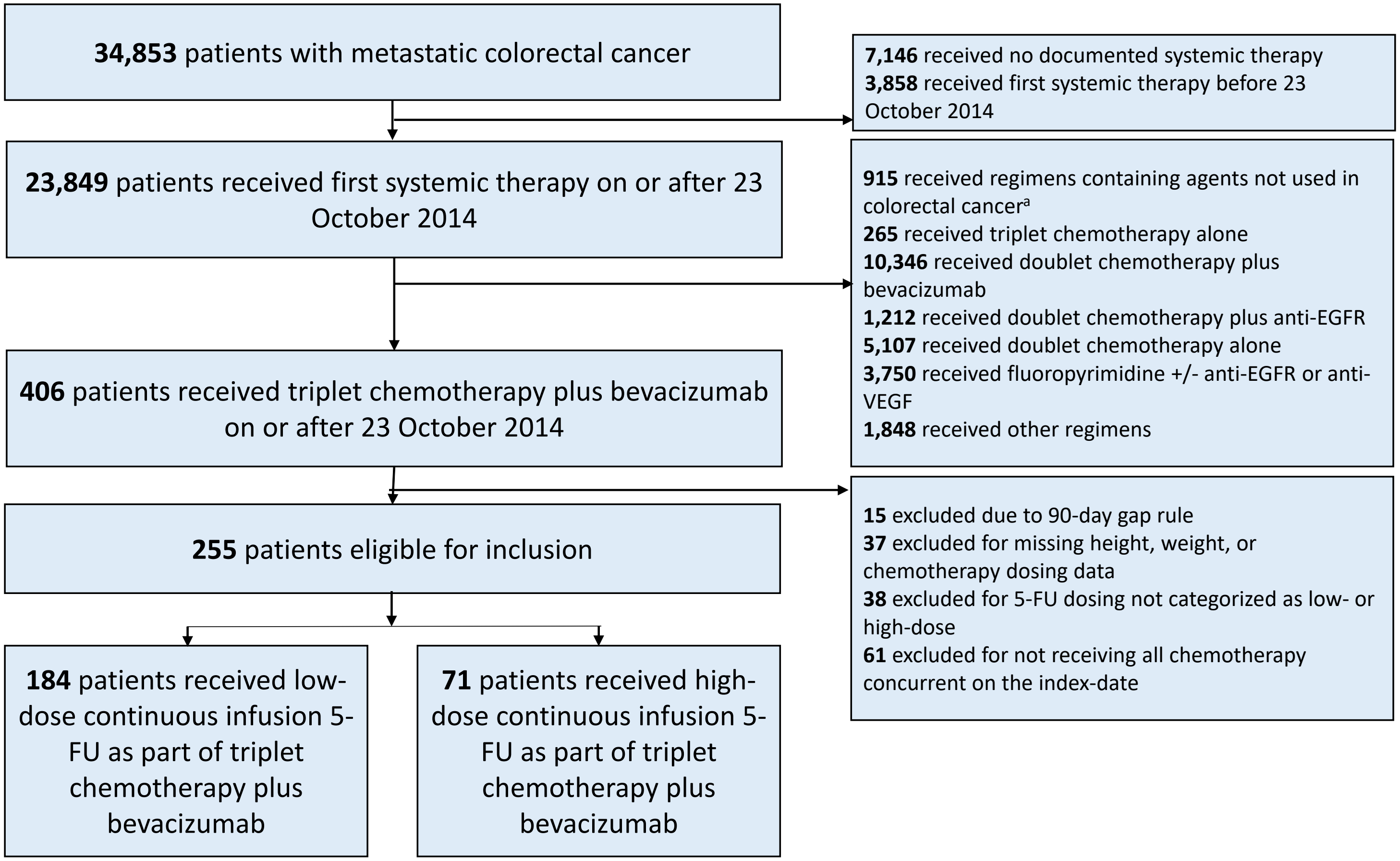

a – Regimens that included both therapies for colorectal cancer and hormonal therapies for breast or prostate cancer were not excluded.

© 2024 Chapin WJ et al. *JAMA Network Open.*

4. eReferences:

1. Luhn P, Kuk D, Carrigan G, et al. Validation of diagnosis codes to identify side of colon in an electronic health record registry. *BMC Med Res Methodol*. 2019;19(1):177. doi:10.1186/s12874-019-0824-7

2. Johnson PJ, Berhane S, Kagebayashi C, et al. Assessment of liver function in patients with hepatocellular carcinoma: a new evidence-based approach-the ALBI grade. *J Clin Oncol Off J Am Soc Clin Oncol*. 2015;33(6):550-558. doi:10.1200/JCO.2014.57.9151

3. Lunt M. Propensity Analysis in Stata Revision: 1. :30.

4. Leyrat C, Seaman SR, White IR, et al. Propensity score analysis with partially observed covariates: How should multiple imputation be used? *Stat Methods Med Res*. 2019;28(1):3-19. doi:10.1177/0962280217713032

© 2024 Chapin WJ et al. *JAMA Network Open.*
