# Supplementary material for: Low- vs High-Dose 5-FU in Triplet Chemotherapy Plus Bevacizumab for Patients With Colorectal Cancer
Source: JAMA Netw Open. 2024 Jul 31;7(7):e2424855. doi: 10.1001/jamanetworkopen.2024.24855 (PMC11292450; doi:10.1001/jamanetworkopen.2024.24855)
Supplement: Supplement 2. — Data Sharing Statement [file jamanetwopen-e2424855-s002.pdf]

Data Sharing Statement

Chapin. Low- vs High-Dose 5-FU in Triplet Chemotherapy Plus Bevacizumab for Patients With Colorectal Cancer. *JAMA Netw Open*. Published July 31, 2024.
doi:10.1001/jamanetworkopen.2024.24855

Data
Data available: No

Additional Information
Explanation for why data not available: Flatiron Health will evaluate all requests to access the data, and if granted, will share the data following completion of a formal data access agreement.
